# Mechanophenotyping of B16 Melanoma Cell Variants for the Assessment of the Efficacy of (-)-Epigallocatechin Gallate Treatment Using a Tapered Microfluidic Device

**DOI:** 10.3390/mi10030207

**Published:** 2019-03-25

**Authors:** Masanori Nakamura, Daichi Ono, Shukei Sugita

**Affiliations:** Department of Electrical and Mechanical Engineering, Nagoya Institute of Technology, Nagoya 466-8555, Japan; 30413043@stn.nitech.ac.jp (D.O.); sugita.shukei@nitech.ac.jp (S.S.)

**Keywords:** microfluidics, mechanophenotyping, cancer, metastatic potential

## Abstract

Metastatic cancer cells are known to have a smaller cell stiffness than healthy cells because the small stiffness is beneficial for passing through the extracellular matrix when the cancer cells instigate a metastatic process. Here we developed a simple and handy microfluidic system to assess metastatic capacity of the cancer cells from a mechanical point of view. A tapered microchannel was devised through which a cell was compressed while passing. Two metastasis B16 melanoma variants (B16-F1 and B16-F10) were examined. The shape recovery process of the cell from a compressed state was evaluated with the Kelvin–Voigt model. The results demonstrated that the B16-F10 cells showed a larger time constant of shape recovery than B16-F1 cells, although no significant difference in the initial strain was observed between B16-F1 cells and B16-F10 cells. We further investigated effects of catechin on the cell deformability and found that the deformability of B16-F10 cells was significantly decreased and became equivalent to that of untreated B16-F1 cells. These results addressed the utility of the present system to handily but roughly assess the metastatic capacity of cancer cells and to investigate drug efficacy on the metastatic capacity.

## 1. Introduction

Metastasis is the spread of cancer cells from the primary site of origin to other sites of the body. When cancer cells metastasize, some cells known as circulating tumor cells (CTC), penetrate the endothelium and the basement membrane and pass through a tiny gap in the extracellular matrix [1,2,3]. It is therefore thought that having smaller stiffness is beneficial for cancer cells to instigate the metastatic process [4]. In support of this, only few studies show that cancer cells are stiffer, and a large majority of experiments indicate that cancerous cells are softer than their benign counterparts and that cellular rigidity decreases with the progression of the disease—as summarized in Aliber et al. [4]—although it remains unclear whether such cell softening is a universal feature [5].

Various tests were conducted to mechanically characterize living cells including cancer cells [6,7,8,9,10,11,12,13,14,15]. Mechanical studies on cancer cells and related cancer biology are thoroughly reviewed in Darling et al. [16], Aliber et al. [4], and Chaudhuri et al. [17]. Using atomic force microscopy, Cross et al. [8] demonstrated that metastatic cancer cells taken from the pleural fluids of patients with suspected lung, breast, and pancreas cancer have stiffness as much as 70% smaller than the benign cells. Remmerbach et al. [11] measured the compliance of cells from cell lines and primary samples of healthy donors and cancer patients using a microfluidic optical stretcher. They found that cancer cells were on average 3.5 times more compliant than those of healthy donors. Swaminathan et al. [12] showed that cancer cells with the highest migratory and invasive potential are five times less stiff than cells with the lowest potential. Furthermore, they reported that invasiveness decreased when cell stiffness, by restoring expression of the metastasis suppressor TβRIII/betaglycan, increased. It was also reported that the more motile and metastatic cancer cells were, the softer they were, indicating that nanomechanical stiffness was inversely correlated with the migration potential of the cancer cell [13,14]. These results suggest that the cell stiffness is a reliable quantitative indicator or a good biomarker of migration and the invasive potential of cancer cells. However, the conventional methods of mechanical tests such as atomic force microscopy, optical tweezers, and micropipette aspiration are time-consuming, labor-intensive, and often require difficult manipulation and mastery skills although they give relatively accurate data [18]. Additionally, each method has its own drawback. For instance, atomic force microscopy requires a cell adhering to a basement. Micropipette aspiration involves difficult manipulation and fine adjustment of pressure. In order to use the cell stiffness as a biomarker of the metastatic potential of cancer cells in clinical practice, more viable methods are demanded.

Microfluidic devices have high throughputs in selecting cancer cells and assessing their metastatic functions [19,20,21,22,23,24]. For example, Hou et al. [22] and Khoo et al. [23] established a spiral microfluidic channel to separate CTCs from blood cells. Tse et al. [24] evaluated the mechanical properties of cells sampled from malignant pleural effusions using a crossed microfluidic channel, proposed by Gossett et al. [25]. These studies addressed a great potential of microfluidic techniques to handily characterize the mechanical properties of cancer cells in clinical practice.

Epigallocatechin gallate (EGCG) is a major component of green tea. Taniguchi et al. [26] reported that EGCG inhibited the spontaneous metastasis of B16-F10 cells and B16-BL6 cells to the lungs of mice. EGCG binds to various proteins and both DNA and RNA molecules [27], it also inhibits binding of ligands and tumor promoters to their receptors in the cell membrane, and the receptor signaling pathway of epidermal growth factor (EGF) [28]. EGCG also works as an immune check point inhibitor [29]. EGCG acts on the cell membrane of cancer cells, hardens the cell membrane [30], and suppresses cancer cell migration and invasion [31]. EGCG blocks induction of carcinogenic factors by hardening the cell membrane and inhibits metastasis of cancer cells [32]. Taking these facts together, it is considered that hardening of cancer cells with EGCG could be another way of inhibiting metastasis of cancer cells.

Better and more appropriate cancer therapies, including a choice of drugs (weak or strong) with minimal side effects, could be applied if the metastatic potentials of the circulating tumor cells were easier to evaluate in clinical practice. The aim of the present study is therefore to investigate the mechanical properties of cancer cells, in particular highlighting internal cytoskeletal structures and changes brought by EGCG treatment. A microflow channel of a simple design was used to evaluate cell stiffness. Here we used two cell types that are known to have different metastatic potential and investigated whether these cells can be differentiated from their viscoelastic properties. The same assessment process was also applied to untreated and EGCG-treated cells to see whether effectiveness of EGCG treatment can be detected using the same procedure as used in differentiating the metastatic potential.

## 2. Materials and Methods

### 2.1. Cell Sample

Mouse melanoma cell lines B16-F1 and B16-F10 were used. B16-F1 was obtained by a one-time selective procedure and B16-F10 by a ten-time selective procedure using Fidler’s method [33], meaning that B16-F10 was a select group of cancer cells having greater invasive and metastatic capacity than B16-F1. The characteristics of the cells were reported in Fidler [33], Poste et al. [34], and Nakamura et al. [35]. The cells were cultured in DMEM (05919, Nissui, Tokyo, Japan) containing 10% fetal bovine serum (172012, Sigma-Aldrich, St. Louis, MO, USA) in a humidified atmosphere of 5% of CO_2_ at 37 °C.

Trypsin (25200-056, Gibco, Gaithersburg, MD, USA) was added to the cells that were semi-confluent to detach them from dishes. After the cells were washed with phosphate buffered saline (PBS(-)), cell samples with a concentration of 3–4 × 10^5^ cells/ml were prepared.

### 2.2. Microflow Channel

**Figure 1:**
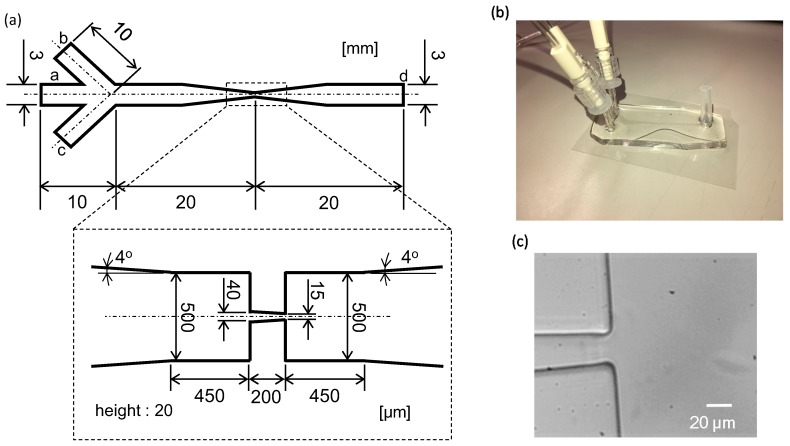
(**a**) Schematic drawing of the microflow channel—a, inlet port for cell flow; b and c, inlet ports for sheath flow; and d, outlet. (**b**) A fabricated microfluidic device and (**c**) a magnified view of the tip of the microflow channel.

Lee et al. [36] and Lima’s group [37,38] used hyperbolic-shaped contraction for the ability to impose a constant strain rate along the centerline of the contraction, as well as to achieve high extensional and shear flows. Although the hyperbolic-shaped contraction was efficient for causing cell deformation, here we used a linearly tapered microflow channel, as shown in Figure 1a. The geometry of the channel is similar to that in TruongVo et al. [39], who also used it to characterize breast cancer cells. The channel has four ports: (a) Inlet for cell flow, (b) and (c) inlets for sheath flow, and (d) outlet. In design, the main flow channel has a taper with an inlet width of 40 μm, an outlet width of 15 μm, and a length of 200 μm. The height of the main channel is 20 μm, providing a rectangular cross-section.

The flow channel was fabricated according to standard photolithography and soft lithography techniques. The negative photoresist pattern was fabricated on a silicon wafer (Matsuzaki, Tokyo, Japan) with SU8- 3050 (Nippon Kayaku, Tokyo, Japan). PDMS prepolymer (Sylgard 184 silicone elastomer kit, Toray Dow Corning, Tokyo, Japan) was poured onto the silicon wafer and baked at 80 °C for 1 h. Plasma treatment was used to chemically bond the PDMS mold to a glass slide with a thickness of 0.12–0.17 mm (C050701, Matsunami, Bellingham, WA, USA). The fabricated microfluidic device and the microscopic image of the tapered part are shown in Figure 1b,c, respectively. In the final product, the width of the tip was 20 μm and the channel height was 32 μm.

### 2.3. Experimental Setup

**Figure 2:**
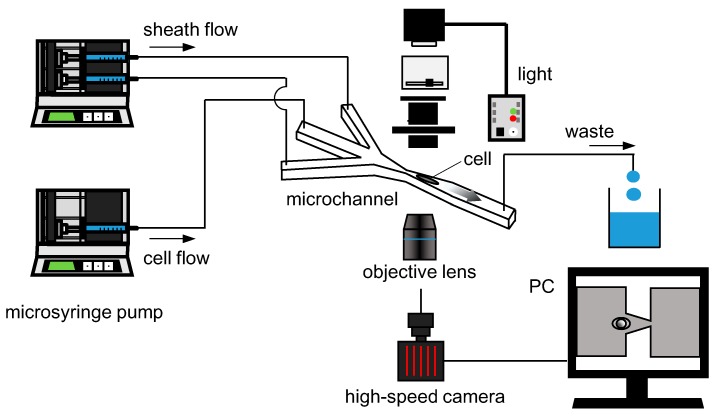
Schematic illustration of the experimental setup. The microflow channel was placed under the microscope and cell deformation was recorded with a high-speed camera.

**Figure 3:**
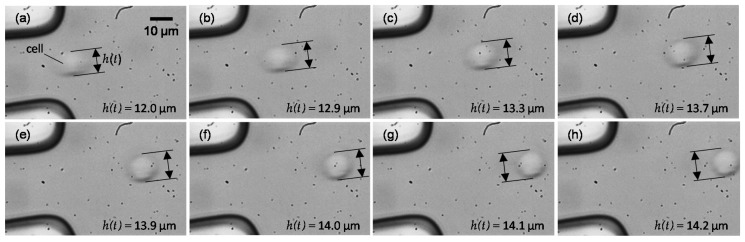
A time series of snapshots of a cell showing how the cell was recovering its shape after it had left the tip of the tapered channel (**a**). The time interval between consecutive snapshots (**b**–**h**) is 20 μs. The scale bar in Figure 3a applies to all images.

Figure 2 provides a schematic illustration of the experimental setup. The experimental setup mainly consists of an inverted microscope (IX-71, Olympus, Tokyo, Japan), a high-speed camera (FASTCAM Mini AX200, Photron, Tokyo, Japan), syringe pumps (KDS-210, KD Scientic, Holliston, MA, USA), and a flow channel. Cells were introduced to the flow channel by one of the syringe pumps. Along with cell flow, sheath flow was also introduced to direct cells to the center of the channel. The total flow rate of the cell flow and sheath flows was set to 66 μL/min that gave approximately 1.5 m/s at the tip of the tapered channel. The ratio of the flowrate between cell flow and sheath flow was 1:6. A cell shape at the tip of the taper and its downstream was recorded with a high-speed camera at a frame rate of 100,000 fps via an objective lens of 60x (N.A. 0.7, LUCPlanFLN 60x, Olympus). The cell height *h*(*t*)—defined as cell length in a direction perpendicular to the flow, exemplified in Figure 3—was measured using image analysis software (ImageJ 1.48v, National Institutes of Health, Bethesda, MD, USA).

### 2.4. Mechanical Characterization of a Cell

Cells leaving the tapered channel are released from compressive forces and gradually recover to their original shape. Here, the compression strain *ε*(*t*) of a cell at time *t* after leaving the tapered channel—was defined by the following formula:

(1) $$\varepsilon \left(t\right)=\frac{h_{\infty }-h\left(t\right)}{h_{\infty }}$$

where *h*(*t*) is the cell height at time *t*, and *h*_∞_ is the cell height in the last frame where the cell is sufficiently far from the tip of the tapered channel.

The recovery process of the cell diameter was expressed with a Kelvin–Voigt model that has a purely viscous damper with a viscosity of *μ* and a purely elastic spring with a spring constant *k* connected in parallel. When a cell leaves the tapered channel, it is released from the compressive force. Under this condition, the compressive strain of the cell, *ε*(*t*), is expressed with the following formula:

(2) $$\varepsilon \left(t\right)=\varepsilon_{0}\exp\left(-\frac{t}{\tau }\right)$$

where *τ* is a time constant of shape recovery and equal to *μ*/*k.*

### 2.5. EGCG Treatment

EGCG is the main polyphenolic constituent of green tea [26]. Reportedly, EGCG inhibits tumor promotion induced by teleocidin in a two-stage carcinogenesis experiment on mouse skin [40] and duodenal carcinogenesis with N-ethyl-N’-nitro-IV-nitrosoguanidine [41]. It is also reported that EGCG inhibits lung colonization of B16-F10 cells and spontaneous metastasis of B16-BL6 cells from the foot to the lung [26]. Clinical trials have demonstrated that green tea catechins including EGCG are effective for cancer prevention [42,43,44,45]. Here, EGCG was used to stiffen cells by following a protocol described in Fujiki and Okuda [46]. An EGCG culture medium of 200 μM/L was prepared by diluting an EGCG/PBS(-) solution of 25 mM/L in DMEM. Cells were cultured with the EGCG culture medium for 4 h. After the culture, the cells were removed from dishes by treatment with trypsin, which was followed by centrifuge and removal of the medium. Finally, EGCG-treated cells were suspended in PBS(-) such that the cell concentration became 3–4 × 10^5^ cells/mL.

### 2.6. Staining

The cell nucleus and actin filaments of B16-F1 and B16-F10 cells were stained. Staining was conducted for both cells that were attached to dishes and cells that were floating. The latter group was prepared by detaching the cells from the dishes with trypsin and leaving them for 30 min at room temperature until the cells become stably spherical. In the following staining processes, the floating cells were always centrifuged at a relative centrifugal force of 17.9 g for 5 min at washing and liquid exchange. First, cells were fixed with 10% neutral buffered formalin for 10 min at room temperature then washed with PBS(-). The cells were then permeated with 0.2% Triton X-100 in PBS(-) for 10 min then washed. This was followed by blocking with 4% albumin from bovine serum (Wako)/PBS(-) solution for 15 min then washing. For staining actin filaments, cells were treated with Alexa Fluor 488 phalloidin, diluted to 1:200 times with PBS(-) in a dark room for 30 min at room temperature. For staining the cell nucleus, cells were treated with Hoechst 33342 diluted to 1:10,000 with 0.2% BSA/PBS(-) solution in a dark room for 30 min at room temperature. Images were obtained using a confocal laser scanning microscope (FV3000, Olympus) with a 60x oil immersion objective lens (N.A. 1.35, UPLSAPO60XO, Olympus). A laser (OBIS, Coherent, Santa Clara, CA, USA) with an excitation wavelength of 488 nm and 405 nm was used to observe actin filaments and cell nuclei, respectively.

### 2.7. Statistical Method

Student’s unpaired *t*-test was used in all statistical analyses. A significance level of 0.05 was used.

## 3. Results

**Figure 4:**
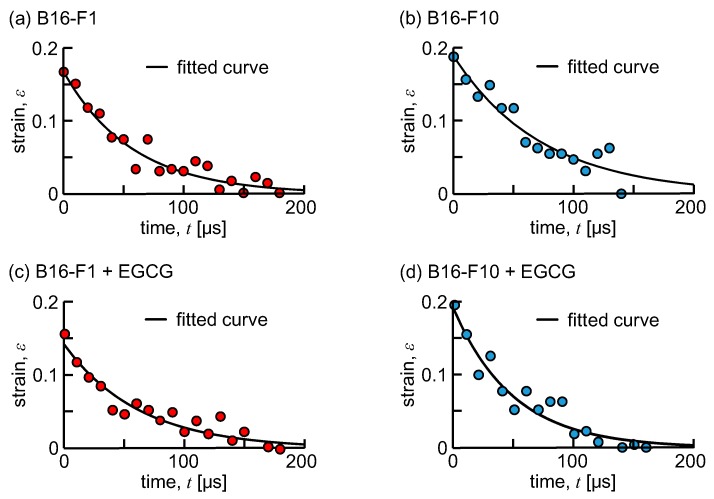
Time variations of the compressive strain of the cell. (**a**) Untreated B16-F1, (**b**) untreated B16-F10, (**c**) epigallocatechin gallate (EGCG)-treated B16-F1, and (**d**) EGCG-treated B16-F10.

Figure 3 shows a series of snapshots of a B16-F10 cell flowing downstream from the tip of the tapered channel. Note that the snapshots in Figure 3 were the ones obtained every two snapshots that were recorded. The cell size at rest was 15.4 ± 1.6 μm for B16-F1 cells and 15.4 ± 1.4 μm for B16-F10 cells, and no statistical difference was found in cell size between them. As seen, the cell that was compressed at the tip gradually recovered its shape to being spherical as it flowed further downstream. A temporal variation of the compressive strain of the cell, *ε*(*t*), is shown in Figure 4 where Figure 4a–d is for untreated B16-F1 cells, untreated B16-F10 cells, EGCG-treated B16-F1 cells, and EGCG-treated B16-F10 cells, respectively. Note that the graphs in Figure 4 are a representative case of each cell and treatment condition. All the figures demonstrate an exponential decrease in *ε*(*t*) as a function of time. Fitting Equation (2) to the data in Figure 4 clearly indicates that a change in the compressive strain could be represented by the Kelvin–Voigt model.

**Figure 5:**
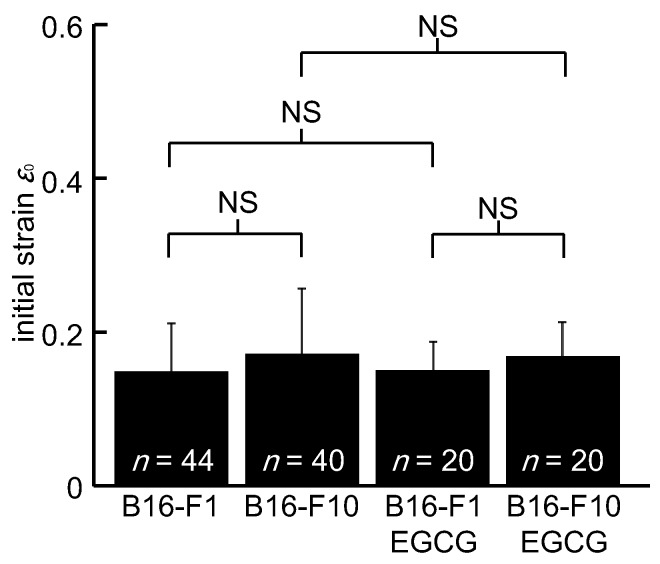
A comparison of the initial compressive strain, *ε*_0_. *n*: Number of cells.

Figure 5 compares the initial compression strain, *ε*_0_, when a cell was at the tip of the taper. The mean ± SD of *ε*_0_ was 0.15 ± 0.06 for untreated B16-F1 cells, 0.17 ± 0.09 for untreated B16-F10 cells, 0.15 ± 0.03 for EGCG-treated B16-F1 cells, and 0.18 ± 0.05 for EGCG-treated B16-F10 cells. No statistical difference was found in *ε*_0_ between any combinations.

**Figure 6:**
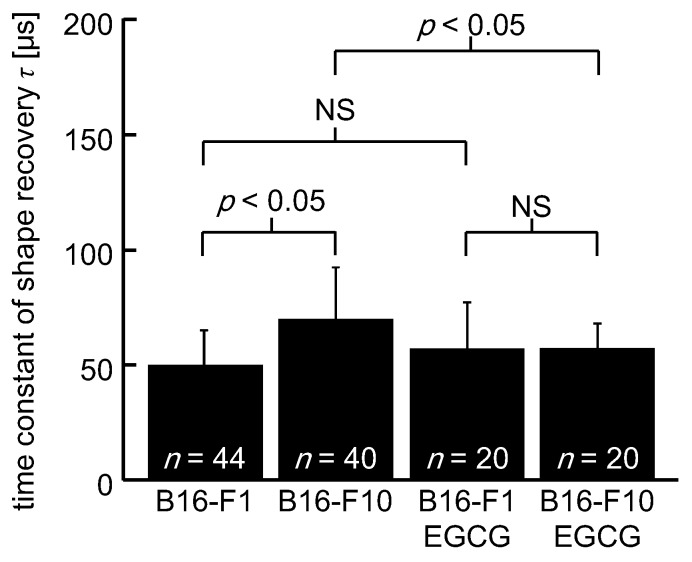
A comparison of the time constant of shape recovery *τ. n*: Number of cells.

A comparison of a time constant of the shape recovery *τ* is presented in Figure 6. The mean ± SD of *τ* was 50 ± 15 μs for untreated B16-F1 cells, 70 ± 23 for untreated B16-F10 cells, 59 ± 22 μs for EGCG-treated B16-F1 cells, and 60 ± 12 μs for EGCG-treated B16-F10 cells. A statistical difference in *τ* was found in a pair of untreated B16-F1 cells vs. untreated B16-F10 cells (*p* < 0.05) and untreated B16-F1 vs EGCG-treated B16-F1 cells (*p* < 0.05), while no statistical difference was noted in a pair of untreated B16-F10 cells vs. EGCG-treated B16-F10 cells and EGCG-treated B16-F1 cells vs EGCG-treated B16-F10 cells.

**Figure 7:**
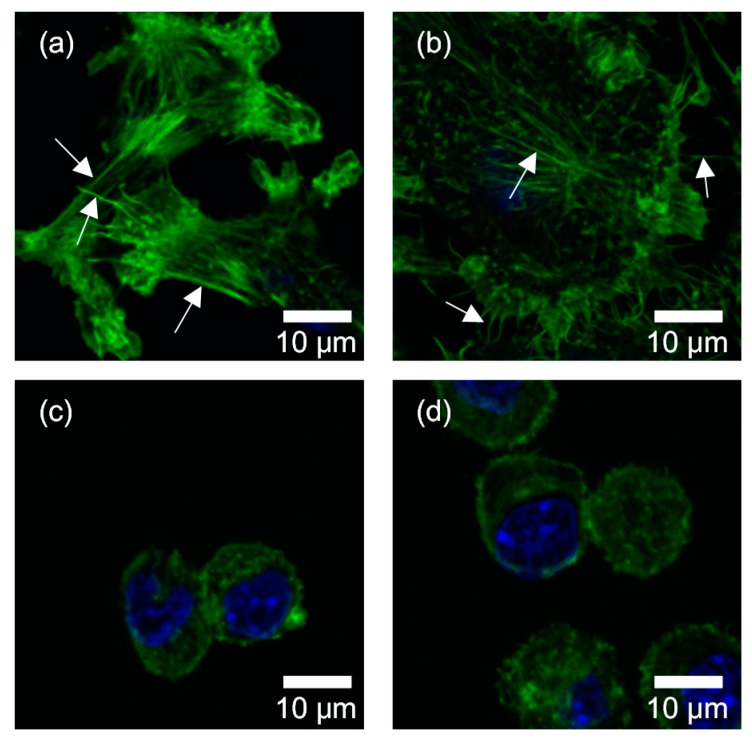
Fluorescent images of actin filaments (green) and nuclei (blue). (**a**) Adhered B16-F1 cells, (**b**) adhered B16-F10 cells, (**c**) floating B16-F1 cells, and (**d**) floating B16-F10 cells. Arrows in (**a**,**b**) indicate actin filaments whose thickness was evaluated.

Figure 7 provides fluorescent images of cellular nuclei and the actin filaments of cells. Figure 7a,b show the cells that remained adhered to dishes, and Figure 7c,d show the cells that were detached from the dishes. Cell lines were B16-F1 for a and c, and B16-F10 for b and d. The detached cells appeared to be spherical, while those adhering to dishes spread with extending processes. In looking at Figure 7a,b, we found that when the cells adhere to dishes, B16-F1 cells had thicker actin filaments than B16-F10 cells, although both cell lines showed fibrous structure of actin filaments. To confirm this perceptual finding, thickness of actin filaments was evaluated with the standard deviation of a Gaussian function fitted to the intensity profile across stress fibers. This is because the spatial resolution of 0.083 μm/pixel in the present images is not fine enough to measure the thickness of actin filaments with a certain accuracy. The evaluation was conducted for three locations for each of the actin filaments arrowed in Figure 7. The results demonstrated 2.15 ± 0.71 pixels for B16-F1 cells and 0.99 ± 0.14 pixels for B16-F10 cells (*p* < 0.05), supporting the perceptual finding of a difference in the thickness. For the cells that were detached from the dishes, the fibrous structure disappeared and no remarkable difference in the structure and amount of actin filaments was noticed between B16-F1 cells and B16-F10 cells.

## 4. Discussion

Microfluidic devices have been used in prior studies to find circulating tumor cells in blood. Recently, Tse et al. [24] invented a microfluidic device of a crossed flow channel at the junction where a cell was deformed by counter striking flows. They successfully classified cells based on cell deformability and took the initiative in diagnosing malignant pleural effusions by microfluidics. Raj et al. [47] fabricated a microfluidic device comprised of multiple parallel microconstrictions. They introduced a theoretical model of cell flow and deformation in the channels and succeeded in quantifying cell elasticity. The present study is situated in part as an extension of these studies. As demonstrated in Figure 6, we found that a time constant of shape recovery could be a useful index to rate the metastatic potentials of cancer cells. Moreover, the time constant could be useful to assess drug-screening applications where biophysical changes occur in cells. The present microfluidic system is totally label-free, which would relieve clinicians from the tangled procedure of labeling and reduce their workload. The microfluidic system proposed here is simple, but its use is not limited to screening of metastatic cells, it has the potential to be used in many areas of medicine other than cancer diagnostics. Although some improvements such as quantification of cell viscoelasticity is necessary, extensive applications of the present system will enable rapid mechanophenotyping of various cells.

Since a tapered portion of the channel was sufficiently long compared to cell size, viscous deformation was assumed to have completed before a cell left the taper. In other words, in the current system, it was considered that the effect of cell viscosity on cell deformation or shape at the tip of the taper was considered to be small and the initial strain *ε*_0_ was determined mostly by cell elasticity. As shown in Figure 5, the initial strain *ε*_0_ of B16-F1 cells was almost the same as that of B16-F10 cells, leading to an assumption that there was no difference in cell elasticity between B16-F1 cells and B16-F10 cells. Moreover, as shown in Figure 6, B16-F10 cells had a significantly larger time constant *τ* than B16-F1. As time constant *τ* is a ratio of the viscosity to the elasticity of a cell, *μ*/*k*, the assumption that there was no difference in cell elasticity between B16-F1 cells and B16-F10 cells indicated that B10-F10 cells had larger cell viscosity than B10-F1 cells. In light of a biological viewpoint that more metastatic and invasive cells should be softer to pass through a narrow gap in extracellular matrix, larger cell viscosity could be unbeneficial for metastatic cells. The biological relevance of larger viscosity for more metastatic cells remains inconclusive and should be clarified in future research.

The width of the flow channel at the exit of the tapered channel was 20 μm. This width might not be small enough to cause large deformation to cells if we consider that the cell size was 15.4 μm in diameter on average. In fact, as shown in Figure 5, we did not find a statistically significant difference in cell stiffness between B16-F1 and B16-F10 cells in the present experimental condition. Two possible reasons were considered. First, loaded cell deformation was not large enough to reflect a difference in cell stiffness. Second, B16-F1 and B16-F10 cells have a comparable level of stiffness in the floating state. Experiments with larger loading by using a device with smaller width will answer this question. At the same time however, narrowing the channel increases the risk of clogging with cells or other debris. Taking into account the practical applications of the proposed channel, clogging has to be avoided. As shown in the present study, a statistically significant difference in the time constant was noticed even with the current width. In this sense, though it was limited to the cell types examined here, the width of 20 μm was considered to be sufficient.

The sheath flow was established in the present flow channel. The sheath flow is necessary as the cell is much smaller than the taper tip and it is important to control cells at a particular position. In this sense, the sheath flow was redundant in the current experiment because the channel width of the taper tip was comparable with cell size.

Although cells flowed along the centerline of the flow channel at the tip, they may have had some rotational motions when they were released into a large pool beyond the taper tip. Due to deformation, cells would have not stayed in the centerline, although they moved downstream by inertia. As a consequence, fluid shear was exerted on cells such that they exhibited rotational motions. Once cells are out of the centerline, they experience a shear-induced lift force that drives them toward channel walls [48]. As deformed cells recover their shape, a time period of the rotation decreases [49], meaning that cells rotate more quickly. However, we did not observe significant cell rotation when looking at cell behaviors in the present experiment. This could be because the experiment’s duration was not long enough to observe cell rotation. As shown in Figure 3 and Figure 4, cell deformations were tracked for only 200 μs in the present experiment. During that period of time, the cell traveled approximately 30 μm, which is 1.5 times as large as a cell size. The cell rotation would have introduced fluctuating errors in cell height, thereby giving errors in the measurement of the time constant of shape recovery. In fact, the time variation of cell height shown in Figure 4a showed fluctuating behaviors. Further experiments are needed to assess the effect of cell rotations on the time constant of shape recovery.

Actin filaments are concerned with the structural strength, shape stability, and deformation behaviors of cells [50,51,52,53]. As seen in Figure 7a,b, in adherent cells, B16-F1 cells appeared to have thicker actin fibers than B16-F10 cells. This observation was consistent with Sadano et al. [54], who found that actin fibers provide cells with mechanical integrity and structurally support the plasma membrane. In this sense, B16-F1 cells that were rich in actin fibers should be stiffer than B16-F10 cells. This speculation was congruent with Watanabe et al. [14], who demonstrated larger elasticity in B16-F1 than B16-F10 using atomic force microscopy. In contrast, the present results demonstrated no difference in cell elasticity between B16-F1 cells and B16-F10 cells. In fact, cell deformation might not be large enough to reflect a difference in elasticity in the present experimental condition. But, assuming that cells were sufficiently deformed, we attribute a discrepancy between Watanabe et al. [14] and the present result to a difference in cell state—cells analyzed in this study were detached from dishes and were suspended in PBS(-). In suspended cells, actins did not have firm fiber structures. The cell detachment from a dish caused depolymerization of filamentous actins (F-actin) into the monomeric globular form of actin (G-actin) as a part of cytoskeletal remodeling. In fact, filamentous structures were not found in floating B16-F1 cells (Figure 7c) anymore, and no remarkable difference was noticed in the structure and amount of actin filaments between B16-F1 cells and B16-F10 cells. These observations indicated that the leveling in cell elasticity of B16-F1 cells and B16-F10 cells was due to the loss of F-actin by cell detachment. Depolymerization of F-actin would have resulted in an increase in the amount of G-actin. Dispersion of G-actin, or a solid particulate phase in a liquid phase of cytoplasm might have resulted in changing the rheological properties of cytoplasm. As shown in Figure 6, the viscosity of B16-F10 cells was larger than that of B16-F1 cells. This would imply that an increase in G-actin provides cytoplasm with its pseudoplastic nature, by which apparent viscosity decreased with increased stress. Future studies should warrant these speculations.

As shown in Figure 5 and Figure 6, for B16-F1 cells, no changes in *ε*_0_ and shape recovery time constant *τ* were observed, regardless of the catechin treatment. In contrast, the shape recovery time constant *τ* of B16-F10 cells was significantly decreased by catechin treatment and was almost the same value as that of B16-F1 cells, indicating that the catechin treatment promoted fast shape recovery of the B16-F10 cells. On the other hand, Figure 5 showed no change in *ε*_0_ of B16-F10 cells between catechin-treated and untreated groups. Since a fluid force is continuously applied to the cells while passing through the tapered part of the flow channel, *ε*_0_ is hardly influenced by the cell viscosity and is thought to be solely determined by cell stiffness. If so, the decrease in the shape recovery time constant *τ* is thought to be due to the decrease in cell viscosity *μ* by catechin treatment. Although the mechanism of how catechin brings a change in the viscosity of cancer cells is unclear, these results suggest that it would be possible to evaluate drug efficacy, at least in highly metastatic cancer cells, using the shape recovery time constant *τ*.

Cells were potentially dead after they passed through microflow channels. A significant loss of cancer cell viability can occur at shear stress levels above 10 Pa [55]. In the present experimental condition, the maximum shear stress of a channel flow was roughly estimated to be 638 Pa under the assumption of the Poiseuille flow. In the study by Zhou et al. [55], cell viability was 83% for the maximum shear stress calculated to be 199 Pa. Their flow channels with smaller maximum shear stress levels reduced cell viability, although a direct application of their results to our study is difficult as their channel is different from the present flow channel in design. However just for cancer cell screening, cells were not necessarily viable after they passed through the flow channel. Cell viability must be cared if filtration, concentration or sorting of cells are included in the scope of application.

Microfluidic techniques and devices offer rapid high throughput in cell mechanophenotyping compared to conventional analytical techniques such as atomic force microscopy (AFM), microaspiration, and optical tweezers [23,24,56,57,58,59]. In AFM, cell samples need to be indented one by one with care, although it allows researchers to map the mechanical properties of a single cell and provide information on cellular structures including cytoskeletal structure. One of the drawbacks of AFM is that it is applicable only to cells that adhere to the base or dish, and thus the use of AFM for floating cancer cells in circulation is not appropriate. Microaspiration and optical tweezers are more conventional approaches for the mechanical characterization of cells. These techniques provide both local and global mechanical properties of cells but are laborious and require partial technical skill. In our experience, it takes more than an hour to measure a few cells. Microfluidic techniques, including that used in the present study, reduce such laboratory workload. A comparison of microfluidic techniques with AFM, microaspiration, and optical tweezers for measuring red blood cell deformability is summarized in Bento et al. [18]. In microfluidic techniques, cells can be continuously scanned once cell flow is supplied. Combined with imaging analysis, cell mechanophenotyping can be automated. As the present system is not equipped with the automatic imaging analysis, cell deformability was assessed manually after the experiment. Yet, indices of the cell deformability such as the time constant were immediately obtained once a cell was identified in a series of recorded images—after some assessments of image quality. Future improvement of imaging analysis will achieve rapid mechanophenotyping of cancer cells.

Spring constant and viscosity coefficient cannot be determined independently with only the present experiment data. Tajikawa et al. [60] and Kohri et al. [61] studied red blood cells using a similar experimental setup, measured the Young’s modulus of red blood cells by a uniaxial tensile test in a separate experiment and estimated the spring constant. This approach however, requires the cell type to be known in advance, and it cannot be applied to this study where it is desired to identify an unknown cell type and evaluate its metastatic potential. Recently, Raj et al. [62] developed a method to estimate the Young’s modulus of the cell. A different approach to estimate the Young’s modulus of floating cells is also given in TruongVo et al. [39], who used a flow channel similar to the present design. If the spring constant *k* can be determined from the Young’s modulus of the cell using the method of Raj et al. [62] and TruongVo et al. [39], the viscosity coefficient *μ* can then be estimated from the shape recovery time constant *τ* and a more detailed analysis of the cell’s mechanical properties can be made.

In the present experiment, the exposure time was 10 μs and the spatial resolution was 0.083 μm/pixel. Because of these conditions, some images were blurred and the boundary of cells was not clear. In the present analysis, cell shape was manually determined. This may have resulted in errors in measuring the cell height and in turn estimating the time constant of shape recovery. If a cell whose diameter at rest is 15.4 μm is imaged and its diameter is measured as 14.8 μm, the compressive strain for this case is approximately 0.039. If a cell diameter is measured two pixels larger, the diameter is quantified as 14.966 μm and the compressive strain is calculated as 0.028. This yields approximately 10% error in the compressive strain. Careful tuning of the exposure time and the use of better spatial resolution will improve the accuracy of the measurement such that an even tiny difference in the mechanical properties between cells is appreciated.

## 5. Conclusions

The present study proposes a method to evaluate metastatic potential by evaluating the viscoelastic properties of cancer cells on a tapered microchannel. The shape recovery time constant *τ* became larger as cancer cells had higher metastatic potential. The results suggested that it would be possible to evaluate the metastatic potential of cancer cells using the shape recovery time constant *τ*. The method is simple, but its use is not limited to screening of metastatic cells. It can be extensively applied to various medical and biological areas other than cancer diagnostics, such as the assessment of drug efficacy. Although further improvements are necessary, the present method will help with rapid mechanophenotyping and screening of metastatic cancer cells in clinical practice.

## References

[1] Orr F.W., Wang H.H., Lafrenie R.M., Scherbarth S., Nance D.M. (2000). Interactions between cancer cells and the endothelium in metastasis. J. Pathol..

[2] Eger A., Mikulits W. (2005). Models of epithelial–mesenchymal transition. Drug Dis. Today Dis. Models.

[3] Chambers A.F., Groom A.C., MacDonald I.C. (2002). Dissemination and growth of cancer cells in metastatic sites. Nat. Rev. Cancer.

[4] Alibert C., Goud B., Manneville J.B. (2017). Are cancer cells really softer than normal cells?. Biol. Cell.

[5] Jonietz E. (2012). Mechanics: The forces of cancer. Nature.

[6] Binnig G., Quate C.F., Gerber C. (1986). Atomic force microscope. Phys. Rev. Lett..

[7] Hochmuth R.M. (2000). Micropipette aspiration of living cells. J. Biomech..

[8] Cross S.E., Jin Y.S., Rao J.Y., Gimzewski J.K. (2007). Nanomechanical analysis of cells from cancer patients. Nat. Nanotechnol..

[9] Suresh S. (2007). Nanomedicine: Elastic clues in cancer detection. Nat. Nanotechnol..

[10] Zhang H., Liu K., Soc J.R. (2008). Optical tweezers for single cells. J. R. Soc. Interface.

[11] Remmerbach T.W., Wottawah F., Dietrich J., Lincoln B., Wittekind C., Guck J. (2009). Oral cancer diagnosis by mechanical phenotyping. Cancer Res..

[12] Swaminathan V., Mythereye K., O’Brien E.T., Berchuck A., Blobe G.C., Superfine R. (2011). Mechanical stiffness grades metastatic potential in patient tumor cells and in cancer cell line. Cancer Res..

[13] Plodinec M., Loparic M., Monnier C.A., Obermann E.C., Zanetti-Dallenbach R., Oertle P., Hyotyla J.T., Aebi U., Bentires-Alj M., Lim R.Y.H. (2012). The nanomechanical signature of breast cancer. Nat. Nanotechnol..

[14] Watanabe T., Kuramochi H., Takahashi A., Imai K., Katsuta N., Nakayama T., Fujiki H., Suganuma M. (2012). Higher cell stiffness indicating lower metastatic potential in B16 melanoma cell variants and in (-)-epigallocatechin gallate-treated cells. J. Cancer Res. Clin. Oncol..

[15] Hayashi K., Iwata M. (2015). Stiffness of cancer cells measured with an AFM indentation method. J. Mech. Behav. Biomed. Mater..

[16] Darling E.M., Di Carlo D. (2015). High-throughput assessment of cellular mechanical properties. Annu. Rev. Biomed. Eng..

[17] Chaudhuri P.K., Low B.C., Lim C.T. (2018). Mechanobiology of tumor growth. Chem. Rev..

[18] Bento D., Rodrigues R.O., Faustino V., Pinho D., Fernandes C.S., Pereira A.I., Garcia V., Miranda J.M., Lima R. (2018). Deformation of red blood cells, air bubbles, and droplets in microfluidic devices: Flow visualizations and measurements. Micromachines.

[19] Tan S.J., Yobas L., Lee G.Y.H., Ong C.N., Lim C.T. (2009). Microdevice for the isolation and enumeration of cancer cells from blood. Biomed. Microdevices.

[20] Chen J., Li J., Sun Y. (2012). Microfluidic approaches for cancer cell detection, characterization, and separation. Lab Chip.

[21] Ma Y.H.V., Middleton K., You L., Sun Y. (2018). A review of microfluidic approaches for investigating cancer extravasation during metastasis. Microsys. Nanoeng..

[22] Hou H.W., Warkiani M.E., Khoo B.L., Li Z.R., Soo R.A., Tan D.S.W., Lim W.T., Han J., Bhagat A.A.S., Lim C.T. (2013). Isolation and retrieval of circulating tumor cells using centrifugal forces. Sci. Rep..

[23] Khoo B.L., Warkiani M.E., Tan D.S.W., Bhagat A.A.S., Irwin D., Lau D.P., Lim A.S.T., Lim K.H., Krisna S.S., Lim W.T. (2014). Clinical validation of an ultra high-throughput spiral microfluidics for the detection and enrichment of viable circulating tumor cells. PLoS ONE.

[24] Tse H.T., Gossett D.R., Moon Y.S., Masaeli M., Sohsman M., Ying Y., Mislick K., Adams R.P., Rao J., Di Carlo D. (2013). Quantitative diagnosis of malignant pleural effusions by single-cell mechanophenotyping. Sci. Transl. Med..

[25] Gossett D.R., Henry T.K., Lee S.A., Ying Y., Lindgrenc A.G., Yang O.O., Rao J., Clark A.T., Carlo D.D. (2012). Hydrodynamic stretching of single cells for large population mechanical phenotyping. Proc. Natl. Acad. Sci. USA.

[26] Taniguchi S., Fujiki H., Kobayashi H., Go H., Miyado K., Sadano H., Shimokawa R. (1992). Effect of (-)-epigallocatechin gallate; the main constituent of green tea; on lung metastasis with mouse B16 melanoma cell lines. Cancer Lett..

[27] Kuzuhara T., Sei Y., Yamaguchi K., Suganuma M., Fujiki H. (2006). DNA and RNA as new binding targets of green tea catechins. J. Biol. Chem..

[28] Sah J.F., Balasubramanian S., Eckert R.L., Rorke E.A. (2004). Epigallocatechin-3-gallate inhibits epidermal growth factor receptor signaling pathway. Evidence for direct inhibition of ERK1/2 and AKT kinases. J. Biol. Chem..

[29] Rawangkan A., Wongsirisin P., Namiki K., Iida K., Kobayashi Y., Shimizu Y., Fujiki H., Suganuma M. (2018). Green tea catechin is an alternative immune checkpoint inhibitor that inhibits PD-L1 expression and lung tumor growth. Molecules.

[30] Tsuchiya H., Nagayama M., Tanaka T., Furusawa M., Kashimata M., Takeuchi H. (2002). Membrane-rigidifying effects of anti-cancer dietary factors. Biofactors.

[31] Fang C.Y., Wu C.C., Hsu H.Y., Chuang H.Y., Huang S.Y., Tsai C.H., Chang Y., Tsao G.S.W., Chen C.L., Chen J.Y. (2015). EGCG inhibits proliferation, invasiveness and tumor growth by up-regulation of adhesion molecules, suppression of gelatinases activity, and induction of apoptosis in nasopharyngeal carcinoma cells. Int. J. Mol. Sci..

[32] Takahashi A., Watanabe T., Mondal A., Suzuki K., Kururu-Kanno M., Li Z., Yamazaki T., Fujiki H., Suganuma M. (2014). Mechanism-based inhabitation of cancer metastasis with (-)-epigallocatechin gallate. Biochem. Biophys. Res. Commun..

[33] Fidler I.J. (1973). Selection of successive tumour lines for metastasis. Nat. New Biol..

[34] Poste G., Doll J., Hart I.R., Fidler I.J. (1980). In vitro selection of murine B16 melanoma variants with enhanced tissue-invasive properties. Cancer Res..

[35] Nakamura K., Yoshikawa N., Yamaguchi Y., Kagota S., Shinozuka K., Kunitomo M. (2002). Characterization of mouse melanoma cell lines by their mortal malignancy using an experimental metastatic model. Life Sci..

[36] Lee S.S., Yim Y., Ahn K.H., Lee S.J. (2009). Extensional flow-based assessment of red blood cell deformability using hyperbolic converging microchannel. Biomed. Microdevices.

[37] Yaginuma T., Oliveira M.S.N., Lima R., Ishikawa T., Yamaguchi T. (2013). Human red blood cell behavior under homogeneous extensional flow in a hyperbolic-shaped microchannel. Biomicrofluidics.

[38] Rodrigues R.O., Lopes R., Pinho D., Pereira A.I., Garcia V., Gassmann S., Sousa P.C., Lima R. (2016). In vitro blood flow and cell-free layer in hyperbolic microchannels: Visualizations and measurements. BioChip J..

[39] TruongVo T.N., Kennedy R.M., Chen H., Chen A., Berndt A., Agarwal M., Zhu L., Nakshatri H., Wallace J., Na S. (2017). Microfluidic channel for characterizing normal and breast cancer cells. J. Micromech. Microeng..

[40] Yoshizawa S., Horiuchi T., Fujiki H., Yoshida T., Okuda T., Sugimura T. (1987). Antitumor promoter activity of (-)-epigallocatechin gallate, the main constituent of “tannin” in green tea. Phytother. Res..

[41] Fujita Y., Yamane T., Tanaka M., Kuwata K., Okuzumi J., Takahashi T., Fujiki H., Okuda T. (1989). Inhibitory effect of (-)-epigallocatechin gallate on carcinogenesis with IV-ethyl-IV’-nitro-N-nitrosoguanidine in mouse duodenum. Jpn. J. Cancer Res..

[42] Bettuzzi S., Brausi M., Rizzi F., Castagnetti G., Peracchia G., Corti A. (2006). Chemoprevention of human prostate cancer by oral administration of green tea catechins in volunteers with highgrade prostate intraepithelial neoplasia: A preliminary report from a one-year proof-of-principle study. Cancer Res..

[43] Tsao A.S., Liu D., Martin J., Tang X.M., Lee J.J., El-Naggar A.K., Wistuba I., Culotta K.S., Mao L., Gillenwater A. (2009). Phase II randomized, placebocontrolled trial of green tea extract in patients with high-risk oral premalignant lesions. Cancer Prev. Res..

[44] Singh B.N., Shankar S., Srivastava R.K. (2011). Green tea catechin, epigallocatechin-3-gallate (EGCG): Mechanisms, perspectives and clinical applications. Biochem. Pharmacol..

[45] Yang C.S., Wang X. (2010). Green tea and cancer prevention. Nutr. Cancer.

[46] Fujiki H., Okuda T. (1992). (−)-Epigallocatechin gallate. Drugs Future.

[47] Raj A., Dixit M., Doble M., Sen A.K. (2017). A combined experimental and theoretical approach towards mechanophenotyping of biological cells using a constricted microchannel. Lab Chip.

[48] Zhou J., Papautsky I. (2013). Fundamentals of inertial focusing in microchannels. Lab Chip.

[49] Masaeli M., Sollier E., Amini H., Mao W., Camacho K., Doshi N., Mitragotri S., Alexeev A., Di Carlo D. (2012). Continuous inertial focusing and separation of particles by shape. Phys. Rev. X.

[50] Peeters E.A.G., Bouten C.V.C., Oomens C.W.J., Bader D.L., Snoeckx L.H.E.H., Baajiens F.P.T. (2004). Anisotropic, three-dimensional deformation of single attached cells under compression. Ann. Biomed. Eng..

[51] Hu S., Eberhard L., Chen J., Love J.L., Butler J.P., Fredberg J.J., Whitesides G.M., Wang N. (2004). Mechanical anisotropy of adherent cells probed by a three-dimensional magnetic twisting device. Am. J. Physiol. Cell Physiol..

[52] Kumar S., Mexwell I.Z., Heisterkamp A., Polte T.R., Lele T.P., Salanga M., Mazur E., Ingber D.E. (2006). Viscoelastic retraction of single living stress fibers and its imoact on cell shape, cytoskeletal organization, and extracellular matrix mechanics. Biophys. J..

[53] Titushkin I., Cho M. (2007). Modulation of cellular mechanics during osteongenic differentiation of human mesenchymal stem cells. Biophys. J..

[54] Sadano H., Shimokawa-Kuroki R., Taniguchi S. (1994). Intracellular localization and biochemical function of variant β-Actin, which inhibits metastasis of B16 melanoma. Cancer Res..

[55] Zhou J., Giridhar P.V., Kasper S., Papautsky I. (2014). Modulation of rotation-induced lift force for cell filtration in a low aspect ratio microchannel. Biomicrofluidics.

[56] Liu Z., Huang F., Du J., Shu W., Feng H., Xu X., Cheng Y. (2013). Rapid isolation of cancer cells using microfluidic deterministic lateral displacement structure. Biomicrofluidics.

[57] Du G., Fang Q., den Toonder J.M.J. (2016). Microfluidics for cell-based high throughput screening platformsd—A review. Anal. Chim. Acta.

[58] Jiang J., Zhao H., Shu W., Tian J., Huang Y., Song Y., Wang R., Li E., Slamon D., Hou D. (2017). An integrated microfluidic device for rapid and high-sensitivity analysis of circulating tumor cells. Sci. Rep..

[59] Nivedita N., Garg N., Lee A.P., Papautsky I. (2017). A high throughput microfluidic platform for size-selective enrichment of cell populations in tissue and blood samples. Analyst.

[60] Tajikawa T. (2014). Quantitative evaluation of erythrocyte deformability by using micro-visualization technique—Measurement of time constant of shape recovery process as a visco-elastic specification of each blood cells. J. Vis. Soc. Jpn..

[61] Kohri S., Kato Y., Tajikawa T., Yamamoto Y., Bando K. (2015). Measurement of erythrocyte deformability by uniaxial stretching—Measurement of apparent Young’s modulus and time constant of shape recovering. Trans. Jpn. Soc. Med. Biol. Eng..

[62] Raj A., Sen A.K. (2018). Entry and passage behavior of biological cells in a constricted compliant microchannel. R. Soc. Chem..

